# Towards a Strategy for Reducing Missed Opportunities for Vaccination in Malawi: Implications of a Qualitative Health Facility Assessment

**Published:** 2018-07-02

**Authors:** Joseph C. Okeibunor, Ikechukwu Ogbuanu, Anya Blanche, Kwame Chiwaya, Geoffrey Chirwa, Zorodzai Machekanyanga, Richard Mihigo, Felicitas Zawaira

**Affiliations:** 1 WHO/AFRO, Brazzaville, Congo; 2 WHO/HQ, Geneva, Switzerland; 3 WHO, Lilongwe, Malawi; 4 Ministry of Health, Malawi; 5 WHO/IST East & Southern Africa

**Keywords:** Missed Opportunities for Vaccination, Reduction Strategies, Immunization, Malawi

## Abstract

**Background:**

Missed opportunities for vaccination (MOVs), estimated to be about 32-47% of child healthcare clinic visits in various settings globally, contribute to unfulfilled childhood vaccination coverage targets in the African region.

**Objective:**

We assessed the extent of MOVs, identify local drivers and test interventions to reduce MOVs in Malawi.

**Methods:**

We conducted in-depth and key informant interviews with administrators of district hospitals and officers in charge of community health facilities. Focus group discussions were held with health workers and caregivers of children under 24 months of age who received services from study health facilities in Malawi. Coverage rates were collected from the health facility records

**Results:**

Vaccination is appreciated in the communities, but coverage is generally below targets. In some facilities, reported coverage was less than 50%. Opportunities to provide up-to-date vaccination for children were missed due to lack of awareness and knowledge of health workers and caregivers, attitude and priority of health workers, long waiting time, poor coordination and referral of eligible children by clinicians and nurses and overall lack of a team approach to vaccination perceived as a responsibility of health surveillance assistants. Other notable issues included limited time of caregivers labouring on estate farms, unavailability of vaccines resulting from poorly functioning of cold chain equipment and limited transport and failure to appreciate the impact of MOV on poor immunization coverage.

**Conclusion:**

Simple, low-cost, pragmatic and community-driven interventions that may reduce MOVs and improve vaccine coverage

## Introduction

Although vaccination is widely recognized as the most cost-effective public health investment and despite huge global investments in the past 40 years, coverage rates have plateaued around 70% in the African Region over the past four years^1^–^9^. Both reported coverages from the WHO-UNICEF Joint Reporting Form (JRF) and administrative coverages are used to coverage data on respective countries. Some countries estimated their sub-national and national coverage using administrative and survey data, with similar methods as those of the WHO UNICEF estimates of national immunization coverage confirm this situation. Lack of services, failure to use existing services, failure of service providers to vaccinate people who are using services each suggest different kidns of problems with different kinds of public health response. Anecdotal evidence suggests that missed opportunities for vaccination may be widely prevalent in the Region and that addressing such opportunities may contribute to an increase in vaccination coverage.

A missed opportunity for vaccination (MOV) is defined as an occasion when a child, who is eligible for vaccination, and with no valid contraindication, visits a health service facility and does not receive all recommended vaccines^10^. Persons eligible for vaccination could miss an opportunity when receiving a different vaccine (immunization contact) or when getting treated for an episode of illness (treatment contact). An example of an MOV during an immunization contact is missing an overdue DTP dose when a child attends the clinic or hospital for measles vaccination.

The number of MOVs in some countries is huge, and it is estimated that the current global prevalence of MOV is between 32-47%^11^. The first systematic literature review on the subject of MOV was conducted in 1993 by Hutchins et al., in which they found a mean prevalence of MOV of 41%^11^. That first review led to important changes in national immunization programs to address missed opportunities and was updated 20 years later, in 2013. The 2013 systematic literature review found a similar prevalence of MOV as was found in 1993 (32-47%), in spite of campaigns by national programs to promote immunization and introduction of new and effective vaccines.

The framework for MOV project entails framework for MOVs. Reducing MOVs involves reinforcing training to ensure healthcare personel perform as they should have been trained to do, compared with the expense of developing services where they are lacking or motivating use of services where demand-side barriers to use of existing services are an issue. With relatively very little effort or cost, reducing MOVs can have an important impact on raising coverage of national immunization programmes, because it is about making sure children who are already being reached by health services are vaccinated during regular health service encounters. It is believed that reducing MOVs can contribute towards achieving the Global Vaccine Action Plan (GVAP) goal of 90% national coverage and 80% in every district or equivalent administrative unit, with three doses of DTP-containing vaccines^12^.

A preliminary desk review and analysis of available immunization data was conducted by WHO in 2014. This analysis estimated the potential gains in coverage if MOVs were to be eliminated, and they demonstrated potential gains in DPT3 and MCV1 coverage as well as an increase in the proportion of countries reaching ≥90% (unpublished data). The key points of the argument include that many children make contacts with the health facilities either accompanying their mothers to health facilities or presenting for other non immunization issues. If these children are check for their immunization completeness, it is possible to track and update them according. Further analysis for all countries showed that overall the gains in DTP3 coverage were variable across countries – countries with greater than 90% initial coverage tended to experience more modest gains than those starting at lower coverage levels. Addressing MOVs thus provides an opportunity to increase coverage by ensuring that overdue vaccinations are provided together with due vaccines, and by enhanced integration of treatment and preventive services.

The World Health Organization (WHO) has drafted an updated generic global methodology for assessing MOVs. The original WHO global protocol was published in 1993, while the PAHO version was published in 2013. To ensure that this methodology is relevant to the African context, and to help institutionalize reduction of MOVs in standard healthcare delivery, the Africa Regional Office of the WHO tested the new MOV method in Malawi in 2015.

The updated protocol outlines both quantitative and qualitative survey methodologies to evaluate MOVs as well as explore potential interventions for reduction of the same. Thus in addition to collecting quantitative data on coverage and vaccine behavior, the new methodology uses both focus group discussions and in-depth interview to provide qualitative data. This new method thus enhances triangulation of information from various sources in describing the situation of vaccine uptake and MOV in the health system. The qualitative methods provide contextual meaning to the quantitative data. This paper is the first to outline the experience of using this new methodology and aims to document the lessons learned so far as well as discuss the various perspectives on why eligible children are missed during health service encounters. To aim of the survey was to assess the extent of MOVs, identify local drivers and test interventions to reduce MOVs in Malawi

## Methods

### Study site

The study was located in Malawi, a landlocked country in Southern Africa. It has a land coverage area of about 118,500 square kilometres divided into three regions (north, centre and south), with a total of 28 administrative districts. The projected population for 2015 was 3.5 million, with 18% or 2.936 million of this comprising of children <5 years. The vital statistics reveal under-five and maternal mortality rates of 112/1000 live births and 675/100,000 live births.

In Malawi, immunization is part of the essential health package delivered mostly by health surveillance assistants (HSAs). In 2010 the national pentavalent coverage reached 93% and all the districts reached >80% coverage. However, the dropout rate between the first and third doses of the pentavalent vaccine was high (18%), and the proportion of fully immunized children was 80.9% (less than the programmatic goal of ≥90%). It is possible that some of the dropouts may be due to missed opportunities for vaccination. A recent preliminary analysis of immunization data revealed that Malawi could increase DPT3 coverage to 96% with the reduction of MOVs. This study was piloted in Malawi to explore this concept.

### Assessment design

The qualitative study was nested within the overall study strategy across 10 districts in three regions. The study was exploratory and adopted a cross-sectional approach using qualitative methods of inquiry, based on in-depth interviews (IDI) and focus group discussion (FGD) designs. It was designed to allow a description and analysis of the perspectives of community members and their health workers on missed opportunities for vaccination. The community perspective of missed opportunities for vaccination is view from a broader viewpoint that recognizes an interaction among a number of variables, including socio-demographic realities of the people as well as their experiences with the health and immunization systems, health problems and relevant interventions. The primary unit of analysis was children between the ages of 0 – 11 months and 12–23 months who attend a clinic in the selected districts on the day of the assessment. However, information on the immunization status of these children was obtained from their parents, health workers, administrators and community members.

### Sample selection

**Figure 1:**
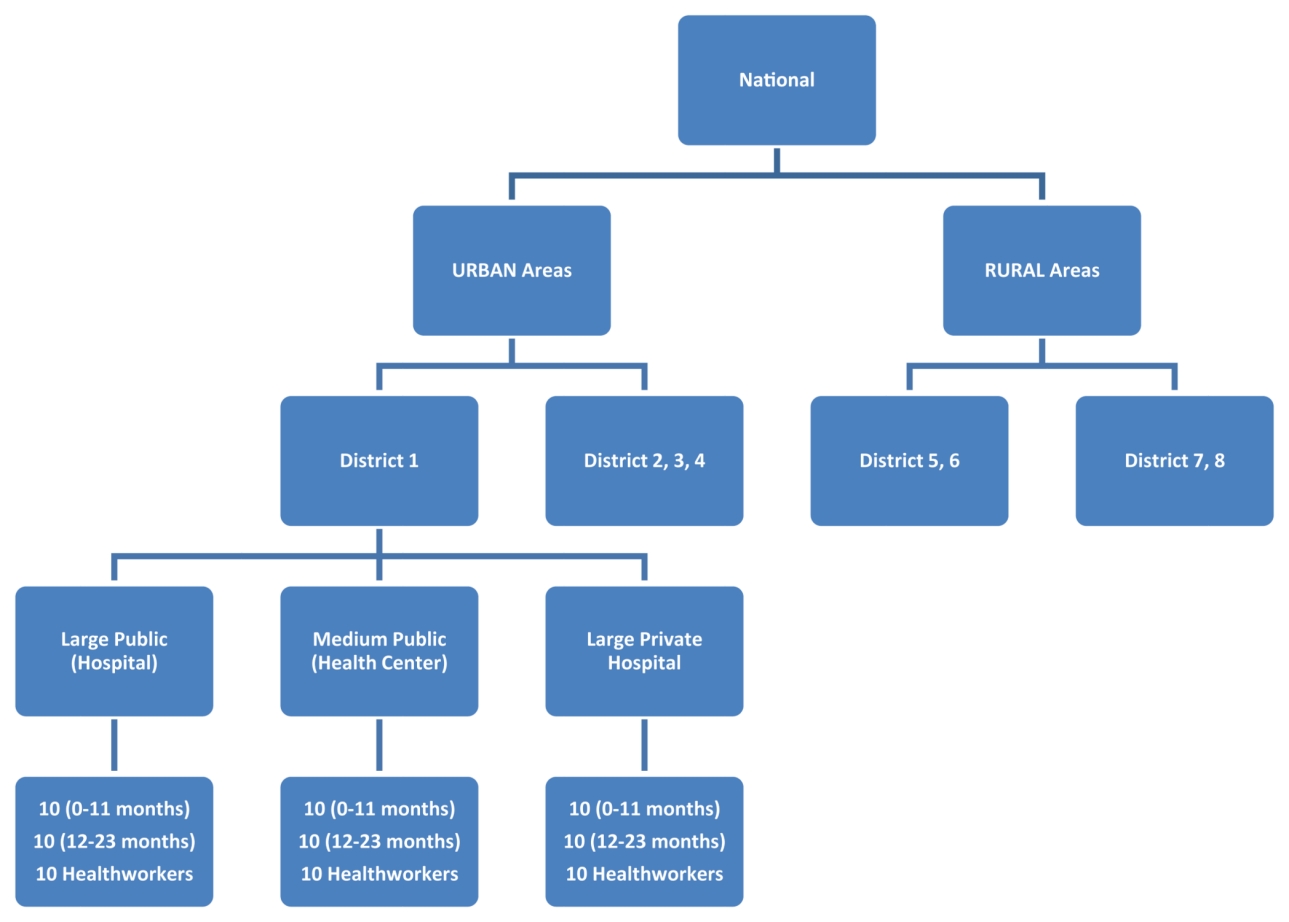
MOV national sampling schematic (Total N=480 children and 240 Health workers)

A simplified sampling mechanism was used as the results are not intended to be nationally representative. At the national level, the study team selected 10 districts for the assessment (Figure1). Within each district, the team also pre-selected three health facilities for the assessment ensuring the inclusion of public and private as well as big and smaller facilities. The selection of health facilities followed a clustering of health facilities into public and private as well as big and small. One health facility was randomly selected from the big or small groups. Where the big is public and one private big health facility was selected in addition. The selection of districts was planned to cover the wide range of geographic and service experiences in the country (including public/private, rural/urban, the size of the facility, and performance level of each district/facility).

At one health facility in each district, about 10 mothers or caregivers were identified after they had received the health services that brought them to the clinic. Each potential participant was pre-screened on age only, and FGD sessions were held with them. FGD sessions were also held with a group of about 5-10 health care workers. We administered an unstructured IDI to the health administrators at the health facility or district level.

### Instruments and method of data collection

Focus group discussion and in-depth interview guides were used for data collection. The study explored the common health problems affecting children in the communities; ways children are protected against such health problems; vaccination issues in the communities, including general status of vaccination of children in the communities and steps taken to ensure children are up-to-date with vaccination in the communities. Discussion and interview sessions were informal and in a relaxed atmosphere.

The FGD and IDI guides were pretested for sensitivity. The pre-testing also provided an opportunity for giving targeted orientation on the methods and objectives of the study to the data collectors. The FGD and IDI sessions lasted an average of 45 and 30 minutes respectively. The principle of homogeneity was maintained during the FGD session, by ensuring same sex groups, particularly in the FGD with health workers. The FGD and IDI sessions were facilitated by trained field assistants with previous experience in the conduct of FGD and IDI. The sessions were conducted in the language the respective groups were most comfortable with.

### Data analysis

Analysis of the data placed emphasis on the verbatim documentation and description of what was said, with minimal interpretation. In going through the transcripts, phrases with contextual or special connotations were noted and pulled out as illustrative quotes in developing the ethnographic summaries. The analysis commenced during the field work while the data were being reviewed and corrected for accuracy and clarity. The next level of analysis began with the review of the interview and discussion experiences with the trained field assistants who facilitated and recorded interviews and discussions; the aim of this next level of analysis was to obtain their views on the factors that inhibited or animated discussions.

A more detailed analysis began with the social scientist reading the transcript. During the first reading, notes were made of major concepts. A second reading utilized a system of open coding. A re-reading of the texts was done to discern patterns in the ordering and clustering of themes, which provided a guide to the systematic development of themes. This process facilitated triangulation of data from discussions and interviews. It may be useful to mention at this point that prevalence of MOVs = eligible (by age and with no contraindication) /number in need of at least one vaccine^13^.

In understanding the issues involved in getting children up to date with their vaccination schedules, some themes emerged as critical in the research findings. These include perceptions of childhood health problems; protection of children against these common health problems and thirdly how vaccination is implemented in the community. Others were health workers’ attitude to vaccination; the attitude of mothers or caregivers towards vaccination and suggestions on reducing missed opportunities for vaccination. These themes guided the analysis of the results.

### Ethical considerations/human subjects (including consideration for vulnerable populations)

The evaluation protocol was submitted to the Ministry of Health IRB (National Health Sciences Research Committee) for review before commencement. Verbal consent was obtained from all mothers/caregivers of children 0–11 and 12–23 months regarding participation in the study, and mothers/caregivers had the option to refuse participation. Because this is a public health programme assessment and not research, this informal consent procedure was sufficient. The personal risks of participants related to this activity were minimal and related to the disclosure of private and potentially sensitive personal information collected in the process. No personally-identifiable information were recorded during the interviews to protect respondents anonymity.

## Results

### Study participants

In the qualitative arm of the study, 189 persons (both health care personnel and caregivers) were enlisted and participated in the discussions (n=180) and in-depth interviews (n=9). Approximately one-third of this was drawn from each of the participating regions. Respondents for the in-depth interviews were mainly health administrators, including the Chief Medical Officer, Matron or medical officers in charge of health facilities. The FGDs included two main participant categories: mothers or caregivers (comprising mainly of women of children bearing age) and health workers (of both genders and with ages between 28 years and 45 years).

The mothers or caregivers for the FGD participants were drawn from various occupational categories. Some were traders or civil servants, particularly in the urban areas. Others were either unemployed or full-time housewives or small scale farmers.

### Health problems affecting children in the community and their prevention

The discussion, whether in the FGD or IDI sessions, opened with a review of the common health problems facing children <24 months in the communities. A number of health problems were mentioned, though with some variation between the urban and rural communities. The commonly mentioned health problems included malaria, diarrhoea, malnutrition, pneumonia, which according to an administrator of health services interviewed *“are the leading causes of hospitalization in the District. We are hoping that the new vaccines introduced will reduce the number of children who fall ill and sometimes die from vaccine preventable diseases”.* Another health worker interviewed listed similar health problems in addition to meningitis. Interestingly these are the same problems listed by mothers or caregivers.

On protection of children against these diseases, some of the participants in the study, especially the health workers wished for effective vaccines against malaria first and foremost and provision of other vaccines to manage the vaccine-preventable diseases. On current practices to manage the problems, one of the health workers interviewed listed as follows:

“For malaria, we give ITN to <5 years and pregnant women. Also, we give IEC to prevent the diseases. For diarrhea, we give IEC. For pneumonia we give immunization. We also give information on diet to prevent infections [IDI: Health worker, Malawi]”

When probed for vaccination, she said,

“No, we also give immunization for other diseases such as polio, measles, tetanus, whooping cough. Another vaccine we administer is BCG against tuberculosis [IDI: Health worker, Malawi].”

### Awareness and perceptions of vaccination services in the community

All participants, community members and health workers of all categories alike, showed some degree of awareness of how vaccination is conducted in all the communities visited. It was shown that in the urban areas with district health facilities, vaccination is conducted every day including Saturdays. However, as one moves away from the district health facilities, vaccination becomes more of an event that occurs very periodically and on a campaign basis. Sometimes people are informed that there will be outreach vaccination services, but they never get to see the health workers for the promised vaccination.

Discussing the organization of vaccination in Mzimba District Hospital, an administrator of health interviewed said:

“Vaccination is done on a daily basis here. Women come every day. One of the interventions given for children is immunization. It is done on a daily basis except on weekends…. Apart from this hospital, another place they get immunization is Banja La Mtsogolo, an NGO. They can also access immunization through outreach clinics and health centers under our District Hospital.”

Another health worker in Mchinji District said:

“Vaccination in this district is offered by Health Surveillance Assistants (HSA) who are based at the health centres in the community. Also, they offer education for health-seeking behaviour change, hygiene with the main focus on preventive medicine. The district employs and pays salaries for all the HSA personnel for vaccination. All health centres in the district provide immunization daily during the weekdays. The Christian Hospital Association of Malawi (CHAM) runs health facilities and also offers immunization services in addition to the community health centres”.

Despite the popularity of vaccination services in the districts, the level of satisfaction reported by the participants seems rather low. When asked to rate the levels of satisfaction with the vaccination programme in their respective districts and communities, the outcomes were very mixed. A health worker in Mzimba said:

“I would say I am not really satisfied because of the challenges we are facing. The major challenge is distance. Our catchments come from very far. The other challenge is that we are also constrained by poor logistics. We have very dusty roads. The roads become very bad when it rains. All of these reduce our coverage. Because this is a large district, it also affects the coverage of the Northern Region. I don’t think we have done much. We have done a lot but not to the desired performance of 80%. I can’t remember the actual figure now.”

Another health worker had this to say:

“According to immunization data, there was a significant increase in the number of children immunized in Mchinji in 2011-2012; however, it was followed by a disappointing decline in 2013-2014. Currently, there is a steady increase in immunized children….Immunization services have been slowed down by lack of resources such as transportation and isolated pockets of religious objections from members of the Zion Christian Church”

Thus, the performance of the vaccination programme was judged rather poorly. According to one officer-in-charge of a health facility:

“Vaccination is appreciated in the communities, but coverage is generally below targets. In our facility the target is 95% but as you can see here it is mostly <50% for many antigens. It is not very common to find children with up-to-date vaccination status **(IDI: HF in-charge)**”

The caregivers, in various FGD sessions, expressed their dissatisfaction with the way vaccination is conducted in the communities. Some of the typical complaints are covered in the sample quotes below:

“At this facility, vaccinations are given during morning hours only. On market days, we fail to come for vaccinations because we have to go to the market first to sell our produce, and by the time we come back from market they have stopped providing vaccination and they say we are late. It would have been good if a free day was chosen than a market day but still to maintain the morning hours as it is more difficult to come for vaccinations in the afternoon [FGD: caregivers].

….Many times we do forget because there are now many vaccines available; again many times health workers do not explain what they are giving and even the side effects of these vaccines [FGD: caregivers]”

## Challenges

The challenges were numerous and covered a number of issues, including human resources and logistics. Some of the mothers/caregivers of the children highlighted concerns with the attitude of health workers towards them. Others complained of poor logistics for keeping up with clinic appointments; inadequate logistics for reaching communities that are located in very far and difficult terrains and ignorance among the mothers or caregivers (Table 1).

The mothers/caregivers were often worried about the vaccine administration techniques used by some health workers. According to a mother/caregiver in Mchinji community, “s*ometimes when our children get vaccinated, they have lumps formed on the vaccination area. I have a 4-year child who still has a lump on her left leg*”. On the other hand, the curative health workers distinguished between their roles as typical health workers: they would not typically attend to mothers/caregivers, who frequently have to wait long hours for the overwhelmed health surveillance assistants (HSAs) whose statutory function is to vaccinate children. According to the officer in charge of one of the health facilities in Mpendu community, “*health workers believe vaccination is mainly the responsibility of HSAs, so mothers wait a long time for HSAs.”*

Other issues that were highlighted include logistic issues relating to multi-dose vaccine vials, cold chain functioning, and transportation logistics. There was also the problem of poor information sharing as well as poor record-keeping in the facilities, which were reported to make it difficult to track the immunization status of children using the health registers and child health passports. Some caregivers in various FGD sessions also complained of the paid employments. According to one of the participants in FGD with caregivers in Mpendu community, *“Sometimes the owners of the estate where we work want us to work 24 hours, leaving us with no time to take the children for vaccination.”*

## Missed Opportunities for Vaccination

Mothers/caregivers indicated that when the child comes to the health centre or hospital for other services, the health worker rarely checked the health passport for the child’s vaccination status. Health workers are, sometimes, not sure what to do and send children who seem older back as over-aged for vaccination. According to a mother in an FGD session in one of the communities, *“I overheard some HSAs discussing on what to do with a child who had not received any vaccine but was over 6 months due, without a proper check.*”

Due to poor record keeping, health workers may use history from the mother/caregivers to know the immunisation status of their children, which could be biased. Similarly, due to the poor transportation of vaccines, there are stock outs being experienced hence leading to the cancellation of clinics. Mothers come to the health centre, but some are put off due to long wait times. Some health facilities practice the booking system, where they wait for a number of children before opening a vial. According to HF in charge:

“Another reason is the vials. For example, BCG vials contain doses for 20 children. One cannot waste a vial because of a child”

Some clinicians find it difficult to go in search of HSAs when a child needing vaccination is seen. The health officer in charge of the health facilities in Mpendu community stressed that *“sometimes HSAs is committed to other activities so you cannot abandon patients in pursuit of HSAs.”*

The mothers also complained that they were never told by the clinician to go for vaccinations after treatment. It was always the mother’s initiative to take the child for vaccinations.

“My child has finished all her vaccinations and she is now one year and nine months old but I have never been told to go for vaccinations when my child was sick.”

## Suggested Interventions

Following the realities of missed opportunities for vaccination in the communities, participants in this study were invited to make suggestions on interventions that should be put in place to reduce missed opportunities and encourage up-to-date vaccination for children in the communities. The following interventions were suggested.

It was strongly suggested to train and motivate health workers to see vaccination as an integral part of health services. Similarly, improving awareness creation and sensitization of mothers and health workers on implications of MOV and on the need to get children up-to-date with vaccination will help to ameliorate this problem. These measures will address the relative ignorance among the mothers on their right to vaccination and why they must insist on getting their children vaccinated up-to-date, as well as the poor knowledge of health workers on their collective roles and responsibilities, independent of the department in which they work.

It was also suggested to train health worker on a follow up (defaulter tracking) system that could reduce missed opportunities for vaccination and drop out. To ensure the effectiveness of the follow-up system, the participants in the study recommended the development of creating a contact list of care givers that includes phone numbers.

Others suggested interventions include improving vaccine administration and coordination across levels of the health service; making vaccines available in flexible vials to reduce wastage and reduce the impact of the “booking system”. Yet another group suggested provision of adequate supply of vaccines and cold chain and ensuring close stock management; and finally, the health workers suggested the provision of transportation for follow up and regular outreaches.

## Discussion

The successful delivery of childhood vaccination depends on the collective responsibilities of all aspects of the health system including the households and caregivers. The responsibility of the different stakeholders have been poorly understood and managed as shown in this study. Mothers/caregivers are often seen as recipients of the benevolence of the health workers. Among the health workers, the health surveillance assistants (HSAs), who are more like auxiliary health workers, are saddled with all the responsibilities of delivering all vaccines in Malawi. We found that the mainstream health workers and clinicians do not perceive vaccination of children as their responsibility. This silo effect is a cause for concern and explains the poor integration of services and the high proportion of MOVs apparent in this study.

Assessing MOV is thus a useful tool both for the health systems (suppliers) and the caregivers/household (consumers of health commodity). Routine screening for immunization status of children should be undertaken on all eligible children to avert outbreak of vaccine preventable diseases that will exert undue pressure on the health system. To facilitate routine screening of the immunization status of children as they come in contact with health service, every child visiting the health system should be made to carry immunization card irrespective of health status. The health personnel should be made to appreciate the screening for status of immunization of the children a core function of the engagement with the health service.

There is also need to demystify the immunization system and increase confidence in the care givers. For instance the practice of not opening a multidose vial for one child should be discourage. In its place, the EPI policy of opening a multidose vial even for one eligible should be emphaised13. Mothers should be continuously enlightened on their right to vaccination and the benefits of ensuring their children are up to date with their vaccination.

**Figure 2:**
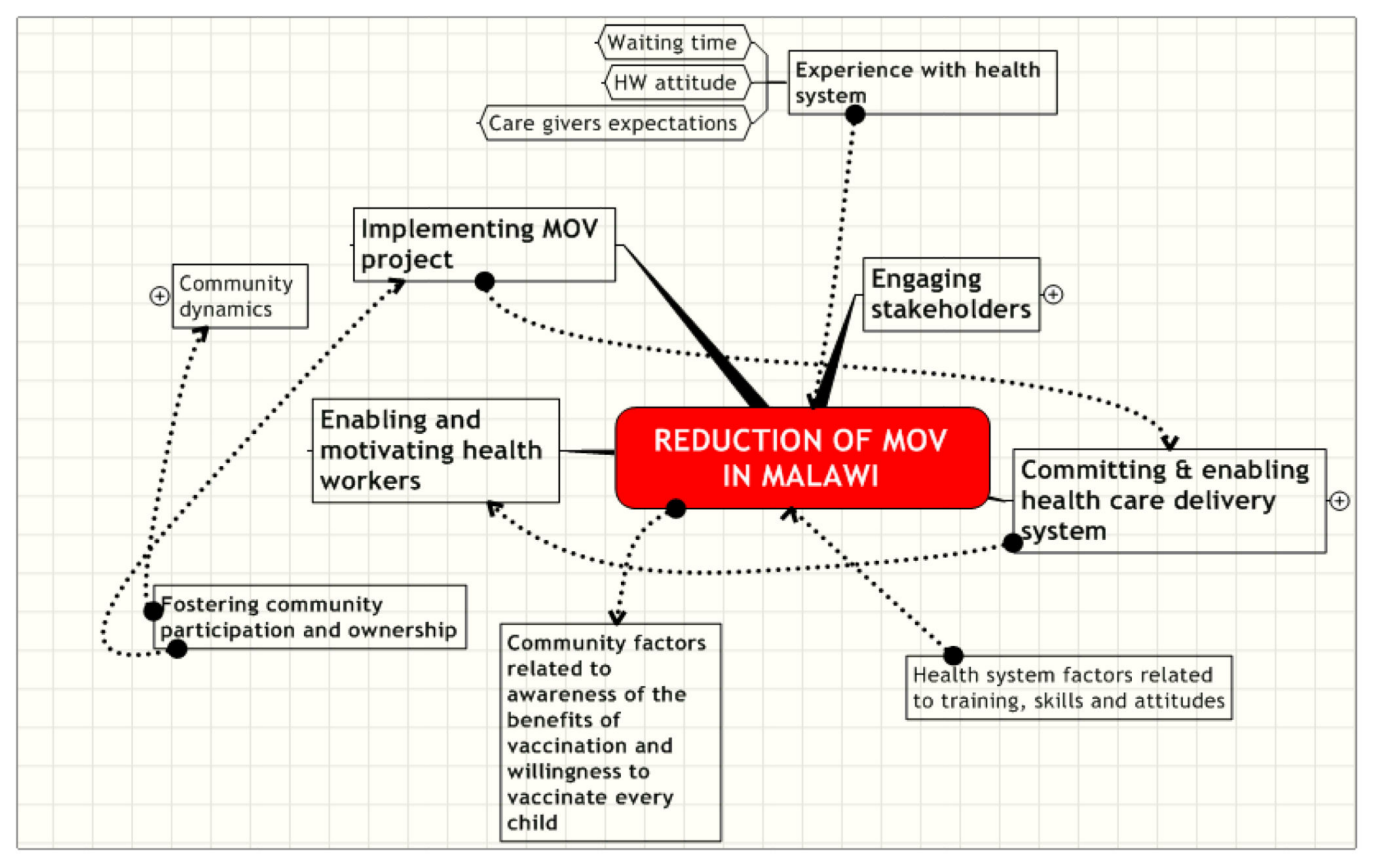
Framework for reducing MOV in Malawi

Given the reality above a framework for understanding missed opportunities for vaccination is shown in Figure 2. In this framework titled the critical factors for MOV implementation in Malawi, there are six major domains of concerns to be appreciated and addressed for the reduction of missed opportunities for vaccination. The domains include the experiences of mothers or caregivers as they interact with the health system; engaging stakeholders; committing and enabling health care delivery system and enabling and motivating health workers. Others are to address health system factors related to training, skills and attitudes and community factors related to awareness of the benefits of vaccination and willingness to vaccinate every child. There is also the need to pay attention to fostering community participation and ownership while implementing the MOV project.

Regarding experience with the health system, the results of this study show that there is a general acceptance of vaccination. However, more positive experiences with the health and vaccination systems will facilitate a reduction in missed opportunities for vaccination in Malawi. Along with improving the experiences mothers or caregivers get when they visit the health facilities to optimize uptake of vaccination services, there is the need for a carefully designed process of carrying the health workers and every other stakeholder along. Currently, reduction of MOVs has not become a national priority According to a health administrator interviewed, “if it is evidence based, we will work to eliminate it.” Thus the health workers and community members and their leaders need sufficient information on the MOV project. A concerted effort to carry all the stakeholders along will result in general agreement with the principles of MOV reduction and a commitment to its reduction.

On the need to ensure enabling health care delivery system, it was observed in this study that vaccination is appreciated. However, there is currently inadequate availability, supply and distribution of vaccines in flexible and manageable vials. Vaccination appears to be a vertical programme in the health system. These issues must be addressed for a successful MOV project in Malawi.

There is also the need to foster community participation and ownership. Although the mothers expressed willingness to ensure their children are up-to-date, they see themselves as being at the mercies of the health workers who dispense the vaccines. If empowered with sufficient information on their rights to vaccination and their responsibilities in ensuring their children are up-to-date, they will demand vaccination. We see empowering community members to get involved in monitoring the performance of the vaccination services for children in their communities as a potential game changer.

The success of MOV reduction will depend on the perceived benefits of the MOV reduction project to the community as well as the commitment of follow up volunteers. There is, therefore, a need for the provision of adequate training, supplies, support and supervision of HSAs, and the full range of staff in the health system to ensure commitment of the health workers to the implementation. Long term commitment to the procurement of vaccines in manageable vials is germane to successful and sustained MOV reduction.

We have shown that missed opportunities for vaccination may account for the poor utilization of health services and that there are simple, low-cost, pragmatic and community-driven interventions that may reduce MOVs and improve vaccine coverage. Although the effectiveness of practical steps for a successful and sustainable implementation are yet to be demonstrated, it is now time for all stakeholders to join hands to address this problem and demonstrate potential impact on vaccination and other services. It is our hope that Malawi and other countries will take this opportunity to empower communities and health workers and improve the overall health and wellbeing of our children.

## Figures and Tables

**Table 1:** Challenges to Immunization listed by health care personnel and child caregivers/mothers

Challenges	Health care personnel	Caregivers/mothers
**Logistics**	▪ Lack of transport for outreach ▪ Bad terrain ▪ Weak cold chain	▪ Distance to health facilities
**Human resources**	▪ Facilities lack sufficient manpower ▪ Division of labour and specialization among health staff ▪ Nurses and clinical staff think immunization is for Health surveillance assistants so do not bother with it	▪ The health worker are not patient enough to check child’s immunization card
**Ignorance**	▪ Not aware of the contribution of MOV to low immunization coverage ▪ If there is a policy on it the health personnel will pay attention to it	▪ Parents did not realize the need to check and update child’s immunization status
**Multi-dose vaccine vials**	▪ The vaccines are packaged in multiple doses and a vial cannot be opened for only one of two children, if the vial is for 12 children because of storage	▪ When we come we are told to return another date when there will be enough children to open the vaccine
**Time factor**		▪ Our plantation employers do not permit us to take our children for immunization Waiting long hours
**Record keeping**	▪ Poor record keeping ▪ No contact of the mothers to ensure reminder calls	
